# Elderly with Varying Extents of Cardiac Disease Show Interindividual Fluctuating Myocardial TRPC6-Immunoreactivity

**DOI:** 10.3390/jcdd10010026

**Published:** 2023-01-09

**Authors:** Jan Michael Federspiel, Jil Gartner, Peter Lipp, Peter Schmidt, Thomas Tschernig

**Affiliations:** 1 Institute for Legal medicine, Saarland University, Campus Homburg, 66421 Homburg, Germany; 2 Institute for Anatomy, Saarland University, Campus Homburg, 66421 Homburg, Germany; 3 Institute for Molecular Cell Biology, Saarland University, Campus Homburg, 66421 Homburg, Germany

**Keywords:** heart, TRPC6, immunohistochemistry, histology, morphology, heart disease

## Abstract

Both particular myocardial locations in the human heart and the canonical transient receptor potential 6 (TRPC6) cation channel have been linked with cardiac pathophysiologies. Thus, the present study mapped TRPC6-protein distribution in select anatomic locations associated with cardiac disease in the context of an orienting pathological assessment. Specimens were obtained from 5 body donors (4 formalin fixation, 1 nitrite pickling salt-ethanol-polyethylene glycol (NEP) fixation; median age 81 years; 2 females) and procured for basic histological stains and TRPC6-immunohistochemistry. The latter was analyzed descriptively regarding distribution and intensity of positive signals. The percentage of positively labelled myocardium was also determined (optical threshold method). Exclusively exploratory statistical analyses were performed. TRPC6-protein was distributed widespread and homogenously within each analyzed sample. TRPC6-immunoreactive myocardial area was comparable regarding the different anatomic regions and sex. A significantly larger area of TRPC6-immunoreactive myocardium was found in the NEP-fixed donor compared to the formalin fixed donors. Two donors with more severe heart disease showed smaller areas of myocardial TRPC6-immunoreactivity overall compared to the other 3 donors. In summary, in the elderly, TRPC6-protein is widely and homogenously distributed, and severe cardiac disease might be associated with less TRPC6-immunoreactive myocardial area. The tissue fixation method represents a potential confounder.

## 1. Introduction

The cation channel transient receptor potential canonical channel 6 (TRPC6) is a candidate factor linking cardiovascular pathophysiologies and calcium [1,2], as it is highly permeable for calcium cations [3]. Thereby, TRPC6 has manifold roles in the healthy and diseased heart, and it is, among others, found in association with remodeling [1], hypertrophy [4], cardiac response to stretch [5], and development of arrhythmia [6]. TRPC6 is widely distributed in the human body, and a systematic TRPC6-mapping has already been performed for other organs and organ systems (e.g., the central nervous system [7]).

A systematic literature search (last database query 8 October 2022; 12:41 pm; database: www.pubmed.ncbi.nlm.nih.gov—National Library of Medicine) identified overall 105 publications regarding TRPC6 and the heart (3 found by “(((location TRPC6) AND (heart)) AND (human)” and 102 identified by “((TRPC6) AND (heart)) AND (human))”). Within these studies, only one article reporting on the distribution of TRPC6-protein within transmural samples of the human heart was identified [8]. Most of the others presented data on animal models (e.g., rat model [9]) or distinct cells (e.g., interstitial cells of human heart valves [10]). The lone study on the TRPC6-protein distribution in the human heart immunohistochemically analyzed TRPC6-protein distribution in the cardiac layers and in rough anatomic parts (i.e., ventricle or atrium) without reporting a standardization of the sampling sites [8]. As such, these samples lack comparability, and a systematic mapping of TRPC6-protein in the human heart has not been reported so far.

Thus, the present study aimed to carry on the immunohistochemical analysis of TRPC6-protein distribution within the human heart by (a) analyzing anatomic regions associated with distinct cardiac pathophysiologies and (b) ensuring comparability of the specimens by standardizing the sampling sites using anatomic landmarks. Additionally, the results were exploratively analyzed in the context of microscopic and macroscopic pathological findings, sex, and the anatomic regions the specimens can be assigned to.

## 2. Materials and Methods

Heart and cardiac tissue of 5 body donors (sex: 2 females, 3 males; age: median 81 years, minimum 75 years, maximum 83 years; postmortem interval (PMI) until fixation: median 87.75 h, minimum 31 h, maximum 100 h) were analyzed. Formalin fixation was used in 4 body donors and nitrite pickling salt-ethanol-polyethylene glycol fixation (NEP) in 1. Details on the fixation of the body donors is provided in Supplement A, while Supplement B summarizes the information on the cause of death according to death certificates.

The following specimens were obtained: (1) right atrial appendage as well as the (2) transition zone of left lower pulmonary vein and left atrium—both associated with atrial fibrillation [11,12]; (3) the head of the left anterior papillary muscle of the mitral valve – chosen because it is frequently affected by fibrosis [13] and cardiomyopathies [14]; (4) the transition zone of fibrous and muscular interventricular septum including the adjacent interleaflet triangle between the right- and non-coronary leaflets of the aortic valve, which is likely to contain parts of the atrioventricular (i.e., bundle of His) [15,16] and the ventricular (i.e., at least left bundle branch) conduction system [15], and is thus associated with conduction disturbances [17]; and the (5) septomarginal trabecula immediately after gathering of both septal limbs containing parts of the right bundle branch [18] by that also frequently a party to conduction disturbances [19].

After these specimens were collected, the hearts underwent organ autopsy. First, the heart size was compared to the respective donor’s fist. A heart was described as enlarged if the heart was larger than the fist. Then, the atria were opened and the tricuspid and mitral valves were inspected. Next, the ascending aorta and pulmonary trunk were trimmed to allow for inspection of the semilunar valves. Then, the hearts were opened following the blood stream through the cardiac chambers. The coronary arteries were opened longitudinally. The left ventricular muscle and the interventricular septum were laminated.

Following previous studies, the severity of coronary atherosclerotic lesions was graded according to the degree of narrowing of the vessel lumen [20,21]. As adapted to the preparation technique described above, coronary stenosis was defined as severe if the narrowed lumen could no longer be transited by scissors while a lumen was still visible, as mild if there was visible reduction of the lumen, but the stenosis was still easily passable by the scissors, and as moderate when the extent of the narrowing of the lumen was between these two other grades. Additionally, the distribution of the coronary heart disease (i.e., 1-, 2-, or 3-vessel disease) was described. Further, it was reported whether the lesions appeared calcified (“crunching” during opening of the vessel, “rock-solid” in palpation) or not.

The tissue was placed in 4% phosphate buffered formalin (Roti-Histofix, Carl Roth, Karlsruhe, Germany) immediately after it was obtained, fixed for 24 h, and then embedded in paraffin (embedding machine MTP, SLEE medical GmbH, Mainz, Germany) and sectioned following the established standard of the anatomic institute involved in this study (thickness 7 µm; microtome: Microm RM 2025, Heidelberg Instruments, Heidelberg, Germany). Specimen orientation during microtomy is described for each sampling site in Supplement C. For the histopathological assessment hematoxylin-eosin-, Masson-Goldner-trichrome-, and alcian-blue-hematoxylin-eosin-stains were applied. The staining protocols are provided in Supplements D to F.

Prior to the study, a proof of specificity was performed using the TRPC6-blocking peptide provided by the manufacturer of the primary antibody (TRPC6 antigen control peptide, #BLP-CC017, Alomone Labs, Jerusalem, Israel). The immunohistochemical labelling of TRPC6 was performed using 3,3′-diaminobenzidine (DAB; SK-4103, Vector Laboratories, Burlingame, CA, USA) as chromogen after application of primary (Ref.: ACC017; Alomone Labs, Jerusalem, Israel, dilution 1:250) and secondary antibodies (Ref.: A10547; Invitrogen, Carlsbad, CA, USA; anti-rabbit coupled with horseradish peroxidase; dilution 1:500) in an indirect immunohistochemistry technique. Thus, brown stain was rated as a positive signal, as counterstain filtered hematoxylin according to Ehrlich was applied for 1 min. Every staining run was accompanied by a negative control. More details regarding the immunohistochemistry can be found in Supplement G.

All stained sections were mounted using Roti-Histokitt II (Carl Roth, Karlsruhe, Germany). Histological assessment was performed using an Olympus BX60 microscope (Olympus, Shinjuku, prefecture Tokyo, Japan), an Olympus D37 camera, and the Olympus “cellSens Dimensions” software (Version 1.15 Build 14760).

First, all specimens were checked for extensive autolysis potentially interfering with the immunohistochemistry by using the standard stains; for the purpose of this study, extensive autolysis was defined as complete disintegration of >15% of the analyzed tissue not allowing for differentiation of the tissue components.

Second, the slides underwent an orienting histopathological assessment. Thereby, endocardial thickening, myocardial fibrosis, fatty degeneration, marked atherosclerosis of small vessels, signs of cardiomyocyte death, and cardiomyocyte hypertrophy were assessed. Myocardial fibrosis and fatty degeneration of the heart were graded based on the affected area of the examined tissue (as presented by Cui and colleagues [22]). A lesion was classified as mild if ≤25% of the analyzed area was affected. Changes comprising >25% and ≤50% of the analyzed tissue were graded as moderate. Severe lesions involved >50% of the assessed area. Marked atherosclerosis of the small vessels was assessed regarding its presence or absence. It was rated as present if atherosclerotic lesions were observed that affected the tunica intima, respectively, the tunica intima and the tunica media, causing remarkable narrowing of the lumen of an intramyocardial vessel without visible connection to a large subepicardial branch at the same time. Anything less severe was marked as an absence of marked atherosclerosis of small vessels. Hypertrophy was, due to varying orientation of the specimens (please compare Supplement C), classified regarding its presence or absence only. Therefore, the grading scheme of Cui and coworkers [22] was adapted such that the presence of fibers as thick as ≥3 erythrocytes was rated as present cardiomyocyte hypertrophy. If only thinner cardiomyocytes were observed, cardiomyocyte hypertrophy was graded as absent. For the same steric reason, the grading of Cui and colleagues for endocardial thickening [22] was adapted and simplified. Endocardial thickening was rated as present if the endocardial layer was thicker than 30 µm in at least 1 site and was absent if the endocardial layer never exceeded 30 µm thickness. Infiltration of granulocytes, presence of contraction band necrosis, and loss of cardiomyocyte nuclei were graded as signs of acute cardiomyocyte death according to recent recommendations [23]. Besides these parameters, the tissues were generally searched for hints of myocarditis, such as infiltration by cells with morphological features of lymphocytes.

Specimens without extensive autolysis were procured to the immunohistochemistry. The immunohistochemistry was first analyzed by applying histomorphological and descriptive analyses by 2 independent examiners. Thereby, distribution, homogeneity, and intensity of TRPC6-positive signals were captured. The both descriptions were integrated to what is presented below. Subsequently to this strongly investigator-dependent analysis, an optical threshold analysis as a more impartial analysis was applied. This method was used to determine the percentage of the TRPC6-immunoreactive myocardial area. A threshold was defined by selection of morphologically non-artificially positively labelled pixels and their chromaticity in an overall strongly stained slide. Based on this threshold, the software calculated the percentage of the examined field of view with at least equal or stronger chromaticity compared to the threshold. The analysis was conducted at 200× magnification and the same threshold was applied to all slides. Per specimen, the analysis was conducted in 5 high-power fields, which were selected by a systematic random sampling approach to avoid duplicate measurements (meandering; selection of every fifth field of view filled by section area through the sample). The average percentages of TRPC6-positively labelled myocardial area per specimen and per heart were calculated. No other components of the cardiac tissues (e.g., subepicardial fatty tissue or endocardium) underwent optical threshold analysis. The area covered by these other tissues was very limited and thus interfered with the systematic random sampling necessary to avoid double measurements and selection bias.

The statistical analysis was exclusively exploratory in its nature. R (version 4.1.3) and RStudio (Version 2022.07.1+554) were used. For visualization, scatter and box plots were employed. Continuous variables were described using median, minimum, and maximum. Discrete variables were described using absolute frequencies. Comparisons in dependent samples (i.e., intraindividual comparisons) were performed using the Wilcoxon rank–sum test. For comparing independent samples (i.e., interindividual comparisons) Kruskal–Wallis-test was applied. For the comparisons, the specimens were grouped by donor, anatomic region (i.e., atrium vs. ventricle, left heart vs. right heart, ventricular free wall vs. interventricular septum, basal vs. apical ventricular location), and sex. In case these tests suggested statistically significant differences in more than dichotomous comparisons, no post-hoc testing was applied as this would result in comparisons of single individuals. Instead, the differences were described. A significance level of alpha = 0.05 was defined, thus *p* < 0.05 was rated as statistically significant.

Supplement H provides an overview of the materials, chemicals/reagents, and software used.

## 3. Results

**Figure 1:**
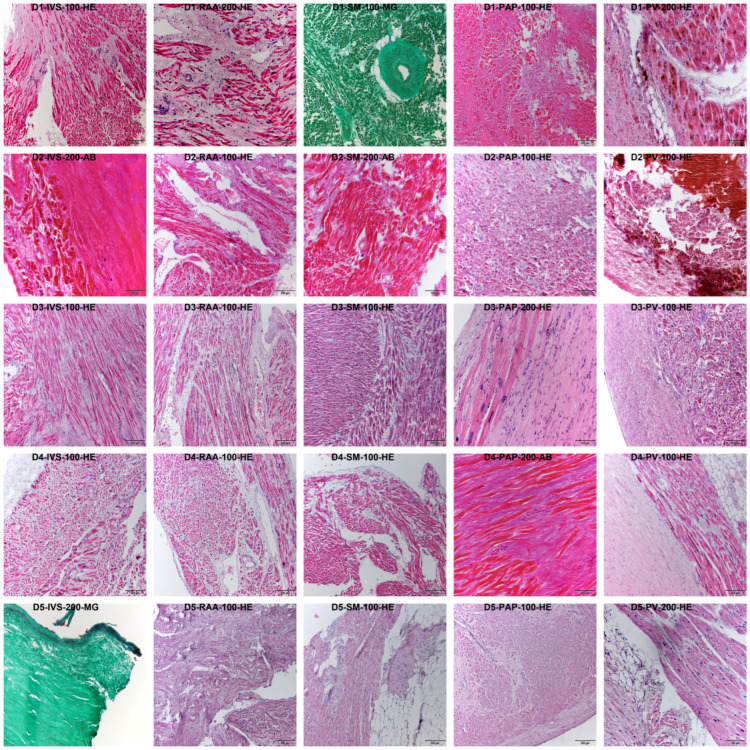
Representative findings in basic histological stains. Per row, one body donor and per column, one sampling site is displayed. Every field has a small heading indicating the body donor (D1—Donor 1, D2—Donor 2, D3—Donor 3, D4—Donor 4, D5—Donor 5), the sampling site (IVS—Interventricular septum, RAA—Right atrial appendage, SM—Septomarginal trabecula, PAP—Anterior papillary muscle, PV—Transition zone of left lower pulmonary vein to the left atrium), the magnification (100–100× magnification, 200–200× magnification), and the basic stain displayed in the picture (AB—Alcian blue, MG—Masson-Goldner-trichrome, HE—Hematoxylin-eosin).

Characteristics of the body donors and the results of the organ autopsy are summarized in Table 1. Table 2 displays the results of the histopathological assessment. Typical histomorphologic findings are shown in Figure 1 (e.g., arteriolosclerosis with surrounding fibrosis—field D1-SM-100-MG; myocardial fibrosis—field D3-SM-100-HE; endocardial thickening—field D4-IVS-100-HE; endocardial fibrosis—field D5-IVS-200-MG; myocardial lipomatosis—field D5-SM-100-HE; fibrosis in the head of the anterior papillary muscle—field D5-PAP-100-HE). None of the analyzed specimens exhibited extensive autolysis. Thus, all samples were procured to immunohistochemistry. Summarizing the gross sectional and microscopic findings, Donor 2 (severe coronary heart disease) and Donor 3 (status post transcatheter aortic valve implantation [TAVI]) exhibited the most severe cardiac disease of the donors analyzed (the remaining 3 donors had mainly moderate coronary heart disease, no exceptional findings in histology).

**Figure 2:**
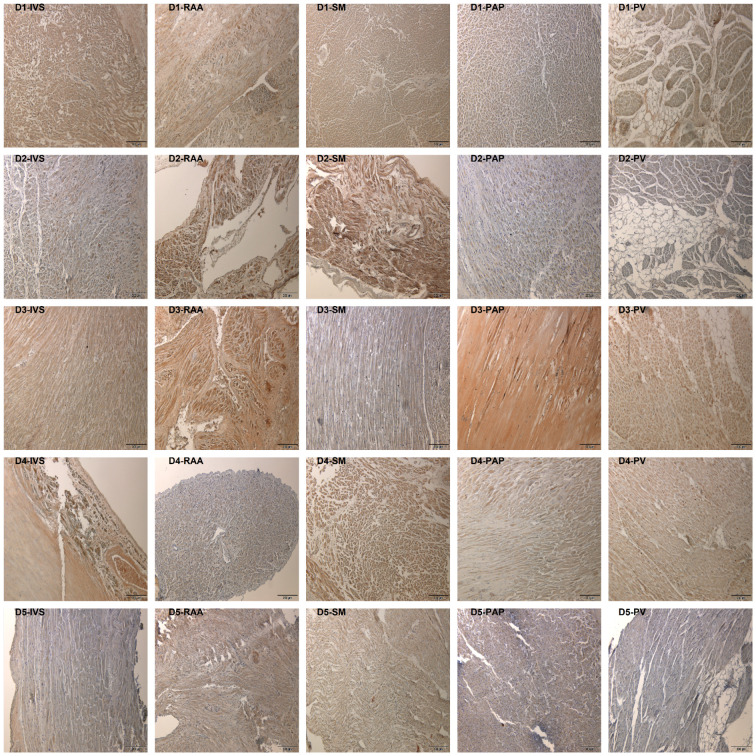
Representative findings in TRPC6-immunohistochemistry. Per row, one body donor and per column, one sampling site is displayed. Every field has a small heading indicating the body donor (D1—Donor 1, D2—Donor 2, D3—Donor 3, D4—Donor 4, D5—Donor 5) and the sampling site (IVS—Interventricular septum, RAA—Right atrial appendage, SM—Septomarginal trabecula, PAP—Anterior papillary muscle, PV—Transition zone of left lower pulmonary vein to the left atrium). All 100× magnification.

**Figure 3:**
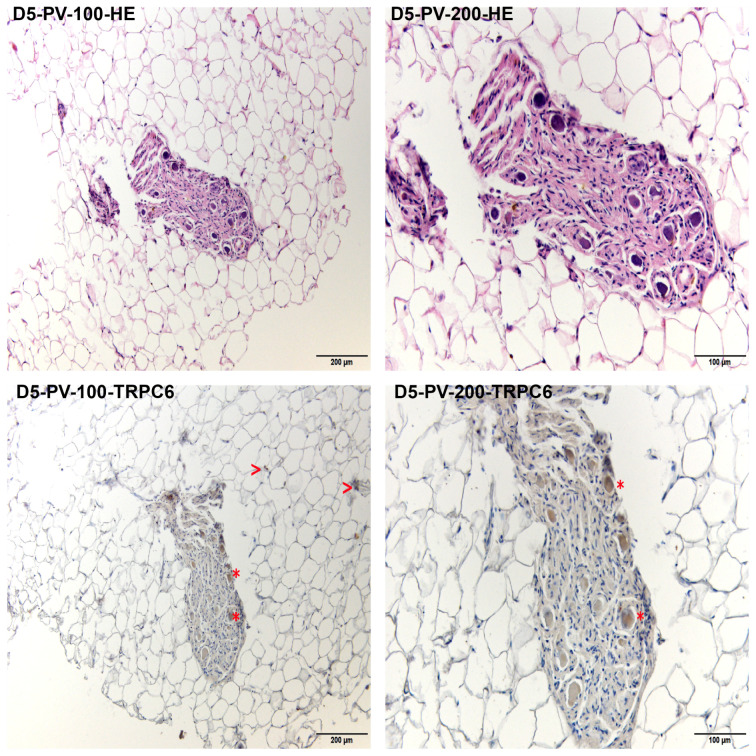
Parasympathetic ganglion in the subepicardial fatty tissue of the left atrium. Every field has a small heading indicating the body donor (D5—Donor 5), the sampling site (PV—Transition zone of left lower pulmonary vein to the left atrium), the magnification (100–100× magnification, 200–200× magnification), and the staining (HE—Hematoxylin-eosin-stain, TRPC6–immunohistochemical labelling of TRPC6). A structure resembling a vegetative ganglion is seen in the center of each field. The two lower images show the repeatedly positive signals (red “*”) found within this structure. Additionally, the surrounding subepicardial fatty tissue presents with scattered positive signals (red ”>”) in areas not affected by wash out of the fat during tissue processing.

The histomorphologic comparison of the TRPC-6 immunohistochemistry (Figure 2) showed varying intensities of the DAB staining comparing different slides (e.g., Figure 2 fields D2-IVS and D3-PAP). These variations are congruent with the differences regarding the overall myocardial TRPC6-immunoreactivity described below. Regardless of the varying signal intensity, the samples showed equal distribution of the TRPC6-protein within the myocardium so that in each analyzed specimen, the myocardium showed homogenous positive signals. Clearly distinguishable from artifacts and compared to the myocardium, more intense positive signals were found in the branches of the coronary circulatory system. The intensity of the positive signals in the myocardium was stronger than in the fibrous tissue, endocardium, subepicardial fat, and epicardium. Compared to the myocardium and coronaries, other components of the heart showed comparable and scarce signals. The only exception was a structure presenting with the characteristics of a parasympathetic ganglion in the epicardial fat of the left atrium of Donor 5 (Figure 3), which also showed strong TRPC6-positivity.

**Figure 4:**
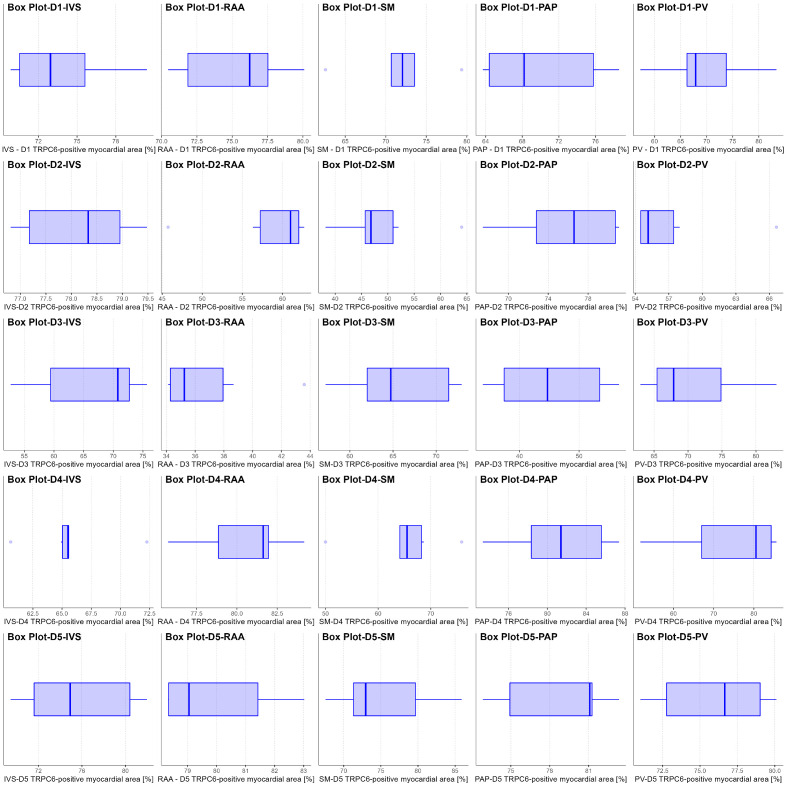
Boxplots showing the distribution of TRPC6-protein positively labelled myocardial area per specimen and donor. Per row, one body donor and per column, one sampling site is displayed. Every field has a small heading indicating the body donor (D1—Donor 1, D2—Donor 2, D3—Donor 3, D4—Donor 4, D5—Donor 5) and the sampling site (IVS—Interventricular septum, RAA—Right atrial appendage, SM—Septomarginal trabecula, PAP—Anterior papillary muscle, PV—Transition zone of left lower pulmonary vein to the left atrium) displayed by the respective box plot (thick line in the box—median; left border of the box—25th percentile; right border of the box—75th percentile; horizontal length of the box—interquartile range; left whisker—25th percentile minus 1.5× interquartile range; right whisker—75th percentile plus 1.5× interquartile range; dots outside the range of both whiskers and the box display outliers).

**Figure 5:**
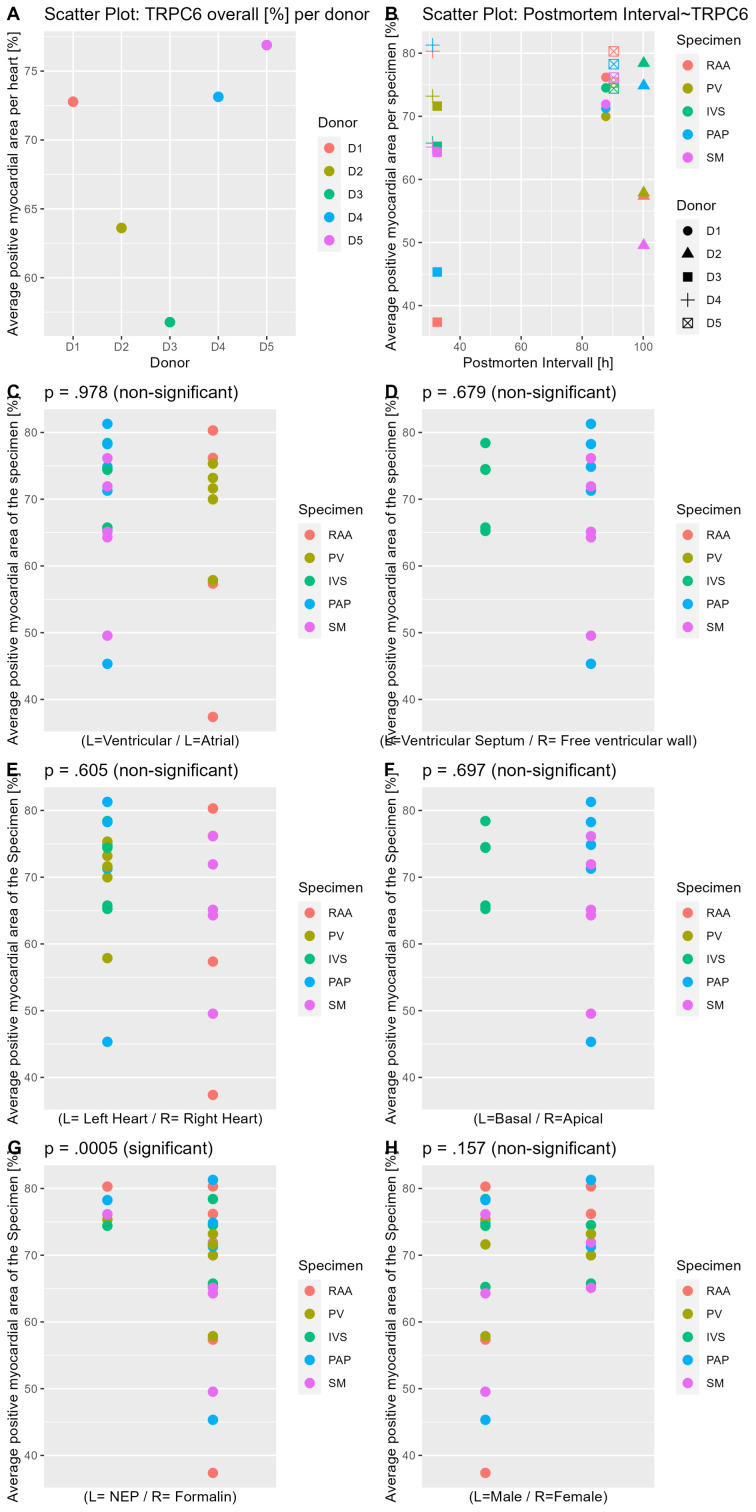
Scatter plots in the comparisons of TRPC6-positively labelled myocardial areas. Both (**A**) and (**B**) display summarizing scatter plots according to the subheading. (**C**–**H**) visualize the different comparative analyses, with the *p*-value of the respective test shown in the subheading of each field and the comparison shown as the label of the x-axis. Legends of colors and shapes are displayed on the right side of each diagram. Abbreviations: D1—Donor 1, D2—Donor 2, D3—Donor 3, D4—Donor 4, D5—Donor 5, IVS—Interventricular septum, RAA—Right atrial appendage, SM—Septomarginal trabecula, PAP—Anterior papillary muscle, PV—Transition zone of left lower pulmonary vein to the left atrium, NEP—Nitrite pickling salt-ethanol-polyethylene glycol fixation.

The results of the optical threshold analysis are shown in Figure 4 and compared in Figure 5. The TRPC6-positive myocardial area of the heart overall (Figure 5A) ranged from a minimum of 56.77% to a maximum of 76.89% with a median of 72.78%. The Kruskal–Wallis-test suggested that this might be a statistically significant difference between the body donors (*p* < 0.001, Figure 5A), and it was noted that the donors with macro- and microscopically more severe heart disease (Donor 2 and Donor 3; for details see Table 1) had less TRPC6-immunoreactive myocardial area overall (both < 65%) compared to the remaining 3 donors (mainly moderate coronary heart disease, all > 72%).

The scatter plot depicting the PMI until fixation and the TRPC6-positively labelled myocardial area shows a randomly appearing distribution of the different spots (Figure 5B).

The samples grouped by anatomic regions were comparable in all instances (Figure 5C–F, *p*-values are given in the figure). In the comparative analysis between the fixation methods, a statistically significant difference was found. The heart after nitrate-ethanol-pickling-salt fixation showed a significantly higher percentage of TRPC6-labelled myocardial area (Figure 5G, *p*-value given in the figure). Males and females were comparable regarding the percentage of TRPC6-positively labelled myocardium (Figure 5H, *p*-value given in the figure).

## 4. Discussion

TRPC6-protein is widely distributed within the human body [24]. Concerning the human heart, initial studies on TRPC6-protein expression in cardiac cells (e.g., the valvular interstitium [10]) or tissues [8] have been published. In accordance with the previous study of TRPC6-protein in human cardiac tissue by Jacobs and colleagues [8], the present study observed a widespread TRPC6-protein distribution within the anatomical regions analyzed, and in addition to those findings the present study has included an analysis of the TRPC6-protein distribution in anatomical areas of distinct clinical relevance in the human heart. The analyzed areas can be sampled reproducibly based on anatomic landmarks. This is the basis for side-to-side comparability of the specimens missing in the earlier study [8]. Additionally, a comprehensive macroscopic and microscopic pathologic analysis based on organ autopsy and histopathological assessment was provided in the present study and is lacking in the so far published study on TRPC6-protein in the human heart [8]. The need for such a background is highlighted by the fact that the macroscopic and microscopic findings in this study differed from the information provided by the death certificates (compare Supplement B). Such a discrepancy is not surprising in light of the documented deficit of quality of death certificates and postmortem examinations in Germany [25].

The organ autopsy showed different extents of cardiac disease (compare Table 1 and Table 2). Additionally, there were hints that these varying degrees of heart disease might be associated with differences in the TRPC6-immunoreactive myocardial area (Figure 5A). That is, the body donors with severe coronary heart disease (Donor 2) and the donor with TAVI (Donor 3) showed the least immunoreactive myocardial area (both < 65%), while the remaining 3 donors, with only moderate coronary heart disease and no valvular intervention, all had more than 72% TRPC6-immunoreactive myocardial area. Although individual donors differed regarding the overall TRPC6-immunoreactive myocardial area, the specimens were comparable among each other and always showed a widespread and homogenous distribution of TRPC6-protein. Additionally, males and females were comparable in terms of TRPC6-immunoreactive myocardial area, despite the sex being a relevant factor in cardiovascular risk stratification [26]. This, in summary, might suggest that the TRPC6-protein functionality and interaction with other components may be even more important in cardiac disease than the bare protein distribution itself, although more severe cardiac disease in the elderly could also be associated with a somehow reduced TRPC6-immunoreactivity in the myocardium.

Interestingly, a significantly larger area of myocardium was labeled TRPC6-protein positive in the tissues obtained from the NEP fixed donor compared to the formalin fixed donors. This might be related to the interference of formalin-induced cross-linking of the proteins [27] with the immunohistochemical method or it may simply be a statistical artifact, as we compared small groups (1 donor vs 4 donors). For clarification, larger series and additional analysis of the protein structure in different fixation methods would be necessary. Moreover, the fixation method applied in the PMI also interferes with the protein structure. So, for example, decomposition causes postmortem protein degradation [28], and liquification of different tissues [29] potentially leading to “dislocation” of the proteins. Based on the apparently random appearing scattering of TRPC6-immunoreactivity plotted against the length of the PMI (Figure 5B), there was no indication of a link between the two in the analyzed samples.

In accordance with the ubiquitous expression of TRPC6 in nervous tissue [7], TRPC6 was found in a parasympathetic ganglion of the left atrium. Thus, together with the involvement of the autonomic nervous system in cardiac diseases [30], we might speculate that TRPC6-protein expression is additionally linked with cardiac disease via the autonomous nervous system also and not by “direct” TRPC6-expression in the myocardium only.

Regarding limitations, it must to be mentioned that with the focus on myocardial locations involved in distinct pathophysiologies, fluctuations of TRPC6 within other parts of the heart might have been overlooked (sampling bias). Additionally, when focusing on those areas involved in distinct arrhythmias, no statement on TRC6 in the context of other cardiac diseases can be made.

The analysis was performed using samples obtained from body donors, and only elderly individuals (age range 75 to 83 years) were included in this study. Despite this limitation, we decided to use tissue from these body donors because: (a) The exact role TRPC6 plays in different cardiovascular diseases and how it is linked with cardiovascular disease is not yet fully understood (see above discussion on the autonomous nervous system). Thus, the use of tissue obtained from routine autopsies of young individuals as “non-failing controls” remains disputable: Does the autonomous activation due to a car accident/violent death alter the TRPC6-analysis? Which efforts must be made to rule out drug abuse as a potential confounder—is a urine analysis enough or is a hair analysis mandatory? And so on. (b) Due to limited data of TRPC6 in human tissue, it is currently not certain whether all the knowledge gained by cell cultures and animal models can be transferred to the human heart. So, although elderly people are likely to be multimorbid and polypharmaceutically treated [31], they are by some means comparable within their group. Nevertheless, with increasing study of TRPC6 in the human heart, further analysis including young and old individuals may become mandatory to determine whether results are widely applicable.

Having analyzed tissue obtained from body donors following fixation, not only the fixation reagent itself but also the fixation technique has to be taken into consideration. A fixation technique applying retrograde perfusion via the femoral artery can flush postmortem blood (clots) in the direction of the heart and the coronaries or possibly into the left ventricular cavity and the coronary arteries. Having said that, some fluctuations of TRPC6-immunoreactivity in the myocardium might be due to partially locally fluctuating fixation intensity. However, significant impairment of the analysis or the immunohistochemical labelling seems unlikely, in light of the similar results in subjective (i.e., descriptive) and objective (i.e., optical threshold) analyses, and because of the exclusion of autolysis interfering with the histomorphologic assessments.

TRPC6 has been embraced as a promising therapeutic target for a variety of diseases including gastric cancer [32], pulmonary hypertension [33], and heart disease [4], and a variety of new substances targeting TRPC6 are being developed (e.g., [32,34]). At the same time, it has been demonstrated that well known drugs such as propranolol can influence at least TRPC6-mediated cellular processes (i.e., TRPC6-mediated Zn^2+^ influx [35]). Having said that, current pharmaceutical treatments might be a potential mediating or confounding factor in the analysis of TRPC6 in elderly humans in general and in the human heart. As the medications information for the body donors in this study was not available, this has to be counted as a limitation of the present study.

The applied methods do not allow for a reliable analysis of TRPC6 on a sub-cellular level. Therefore, besides light microscopy, for example, confocal microscopy, would be necessary because it facilitates the analysis of sub-cellular components [36]. Further studies complementing the mapping of TRPC6-protein distribution on a subcellular level bear the potential to provide further understanding of TRPC6 in cardiac disease. In addition, the present study grossly analyzed the sampled locations. Further, studies are required to analyze the specific sub-structures of the conduction system including the bundle of His and the left and right bundle branches. Serial sections, for example, can be helpful to reliably detect these structures, as was impressively shown by Tawara [37]. Such a somehow “targeted” analysis would again provide further insights in the involvement of TRPC6 in cardiac disease.

By defining the optical threshold in a section with strong DAB stain, falsely high measurement of a positively labelled area was ruled out. Although this carries the potential that staining artifacts are recognized as a correctly labelled area the agreement between the findings in the histomorphologic analysis and the results of the threshold analysis indicate that, such an error is unlikely.

The small case number (*n* = 5) strongly militates statistical power. Additionally, statistical testing is associated with alpha-inflation. So, the observed statistically significant differences must be interpreted with restraint and should mainly serve as hints. Further, it has been shown that sampling different organs from different individuals can severely influence the observed interindividual variance [38]. This is another reason why the findings can only be seen as a hint. Larger series are required to register the impact of the inter- and intraindividual variance and to clearly differentiate between the impact of cardiovascular disease and these variances. 

## 5. Conclusions

This study is the first to systematically analyze TRPC6-protein distribution within comparable samples of the human heart. Thereby, the focus of the study was to provide a somehow “general overview” of how TRPC6-protein is distributed within cardiac locations of known clinical relevance in the context of gross sectional and histological pathological findings. Additionally, the areas of myocardial TRPC6-immunoreactivity of the samples were grouped and compared by sex, body donor, fixation method, and the anatomic locale. TRPC6-protein was found to be ubiquitously present and equally distributed within each sample investigated, and no differences were encountered regarding sex and the anatomic region. There was some indication that severe cardiac disease in the elderly might be associated with a decreased area of TRPC6-immunoreactive myocardium in the analyzed locations. The fixation method of the donor bodies could potentially interfere with the analyses of TRPC6-protein.

## 6. Patents

There are no patents resulting from the work reported in this manuscript.

## Figures and Tables

**Table 1:** Summary of body donor characteristics and gross sectional findings. Abbreviations: NEP—Nitrite pickling salt-ethanol-polyethylene glycol fixation; PMI—Postmortem interval; TAVI—Transcatheter aortic valve implantation.

	Donor 1	Donor 2	Donor 3	Donor 4	Donor 5
**Sex/Age (years)**	Female/76	Male/75	Male/82	Female/83	Male/81
**Fixation / PMI (h)**	Formalin/87.75	Formalin/100.18	Formalin/32.5	Formalin/31	NEP/90.35
**Organ Autopsy**					
**Coronary heart disease**	Moderate 3-vessel disease	Severe 3-vessel disease	Moderate 3-vessel disease	Moderate 3-vessel disease	Moderate 3-vessel disease
**Plaque calcification**	Scattered	Extensive	Extensive	Scattered	Scattered
**Heart size**	Unremarkable	Enlarged	Enlarged	Unremarkable	Enlarged
**Additional findings**	Pericardial tamponade due to Stanford type A dissection	None	Status post TAVI	None	None

**Table 2:** Orienting histopathological findings. Definitions of the different parameters and their grading are provided in the methods section.

	Donor 1	Donor 2	Donor 3	Donor 4	Donor 5
**Myocardial lipomatosis**	Mostly mild. Moderate effect at the septomarginal trabecula.	Mild	Mild	Mild	Mostly mild. Moderate effects at the septomarginal trabecula.
**Marked atherosclerosis of small vessels**	Present	Present	Present	Present	Present
**Myocardial fibrosis**	Mostly mild, marked around small vessels; moderate effects at the papillary muscle	Moderate with severe effects at the papillary muscle and marked around small vessels	Mostly mild, marked around small vessels; moderate effects the papillary muscle	Mostly mild, moderate affection of the muscular interventricular septum	Mostly mild, marked around small vessels; moderate affection of the papillary muscle
**Cardiomyocyte hypertrophy**	Present	Present	Present	Present	Present
**Endocardial thickening**	Present	Present	Present	Present	Present
**Signs of cardiomyocyte death**	Absent	Absent	Absent	Absent	Absent
**Hints for myocarditis**	Absent	Absent	Absent	Absent	Absent

## Data Availability

All data generated or analyzed during this study are included in this published article and its Supplemental Material.

## Supplementary Materials

The following supporting information can be downloaded at: https://www.mdpi.com/article/10.3390/jcdd10010026/s1,

Supplement A—Fixation of the body donors	ii
Supplement B—Cause of death according to death certificate	iv
Supplement C—Orientation of the specimens	v
Supplement D—Protocol hematoxylin-eosin-stain	vii
Supplement E—Protocol alcian-blue-hematoxylin-eosin-stain	viii
Supplement F—Protocol Masson-Goldner-trichrome-stain	x
Supplement G—TRPC6 immunohistochemistry	xii
Supplement H—Material, chemicals, software	xiv
