# Supplementary material for: Elderly with Varying Extents of Cardiac Disease Show Interindividual Fluctuating Myocardial TRPC6-Immunoreactivity
Source: J Cardiovasc Dev Dis. 2023 Jan 9;10(1):26. doi: 10.3390/jcdd10010026 (PMC9861266; doi:10.3390/jcdd10010026)
Supplement: Supplementary file 1 [file jcdd-10-00026-s001.zip › jcdd-2107034-supplementary.pdf]

# - Appendix -

"In elderly severe cardiac disease is associated with less TRPC6-immunoreactivity in clinically relevant anatomic localizations"

Table of content – Appendix

Supplement A – Fixation of the body donors ii

Supplement B – Cause of death according to death certificate iv

Supplement C – Orientation of the specimens v

Supplement D – Protocol hematoxylin-eosin-stain vii

Supplement E – Protocol alcian-blue-hematoxylin-eosin-stain viii

Supplement F – Protocol Masson-Goldner-trichrome-stain x

Supplement G – TRPC6 immunohistochemistry xii

Supplement H – Material, chemicals, software xiv

Supplement A – Fixation of the body donors

Each body donor was perfused with 10 to 15 liter of fixation solution via the right femoral artery with a pressure of 1.5 to 2 bar. Table A.1 depicts the solution used for the nitrite pickling salt-ethanol-polyethylene glycol (NEP) fixation according to Weigner while table A.2 shows the solution used for formalin fixation according to Basler. The NEP-fixation solution was bought as a ready to use solution (for more details please see supplement H).

Table A.1 – NEP fixation according to Weigner

Substance	Quantity
Water	39 liter
Carion	12 l
Formaldehyde	6 l
Lysoformin	2.7 l
Sodium chloride	2400 g
Calcium chloride	600 g
Thymol	12 g

Table A.2 – Formalin fixation according to Basler

Substance	Percent by weight
Ethanol	25 - <35
polyethylene glycol	10 - <25
Formaldehyde	0,1 - <1
Methanol	0,1 - <1

table A.2 continued

demineralized water	Not further specified by manufacturer
nitrite pickling salt	Not further specified by manufacturer

In the upper and lower limbs as well as the gluteal and nuchal region the respective fixation solution was additionally directly injected using 14G cannulas.

Following perfusion and direct injection the corpses were placed in 3 to 5 % formalin solution for 4 months. Afterwards the body were stored in air- and watertight pharopack caskets by a temperature of 14 degrees Celsius.

Supplement B – Cause of death according to death certificates

Table B.1 – Summary causes of death for each body donor

Donor number	Information death certificate
1	• Embolism<br>• Coxarthrosis
2	• Septic shock<br>• Pneumonia<br>• Urinary tract infection
3	• Omission of dialysis in end-stage chronic kidney disease<br>• Progression metastasized prostate cancer<br>• cerebral ischemia<br>• diabetes mellitus
4	• cardiogenic shock<br>• tachyarrhythmia<br>• coronary heart disease
5	• Prostate carcinoma<br>• Liver metastasis<br>• Chronic obstructive pulmonary disease<br>• Occlusive peripheral arterial disease<br>• Cachexia<br>• Anemia

Supplement C – Orientation of the specimens

Table C.1 – Summary of the orientation of the tissue slices

Specimen	Description orientation
Right atrial appendage	The specimen was embedded so that cross sections through the structure were obtained.
Junction of the left lower pulmonary vein with the left atrium	The samples were obtained so that the specimen was a rectangular strip with its long side comprising the end section of the left lower pulmonary vein, its junction with the left atrium, and the adjacent atrial myocardium. The specimen was embedded following the longitudinal axis of the vessel. So, the slides showed a longitudinal section through the aforementioned structures.
Left anterior papillary muscle	In four randomly chosen samples the head of the papillary muscle was cut in half by a longitudinal section. Both pieces were embedded so that the sections showed a longitudinal plane of the structure. The one remaining sample was cut in half by a cross section. The specimen was embedded resulting in cross sections obtained by microtomy.

Table C.1 continued

Proximal interventricular septum	The specimen was obtained as rectangular piece with its long side comprising both – the membranous and the muscular interventricular septum. The tissue was embedded so that longitudinal sections could have been obtained during microtomy.
Septomarginal trabeculation	The specimen was embedded so that microtomy provided cross sections through the structure.

Supplement D – Protocol hematoxylin-eosin-stain

Xylene	5 min
Xylene	5 min
Xylene	5 min
100% 2-Propanol	5 min
100% 2-Propanol	5 min
90% 2-Propanol	5 min
80% 2-Propanol	5 min
Filtered hematoxylin according to Ehrlich	8 min
Distilled water	rinsing
Fluent water	blueing
Distilled water	rinsing
Eosin (0.1% + 2 drops glacial acetic acid)	2.5 min
90% 2-Propanol	2 – 3x dipping
100% 2-Propanol	5 min
100% 2-Propanol	5 min
Xylene	5 min
Xylene	5 min
Xylene	5 min

Supplement E – Protocol alcian-blue-hematoxylin-eosin-stain

Xylene	10 min
Xylene	10 min
Xylene	10 min
100% Ethanol	5 min
100% Ethanol	5 min
90% Ethanol	5 min
80% Ethanol	5 min
70% Ethanol	5 min
Distilled water	5 min
Distilled water	5 min

3% Acetic acid	5 min
Alcian-blue-solution	45 min
Distilled water	1 min
Hematoxylin according to Mayer	10 min
Distilled water	rinsing
Hydrochloric acid-alcohol	differentiation
Water	10 min
Eosin	3 min

Distilled water	rinsing
70% Ethanol	2 – 3x dipping
80% Ethanol	2 – 3x dipping
90% Ethanol	2 – 3x dipping
100% Ethanol	3 min
100% Ethanol	3 min
Xylene	3 min
Xylene	3 min
Xylene	3 min

Supplement F – Protocol Masson-Goldner-trichrome-stain

Xylene	10 min
Xylene	10 min
Xylene	10 min
100% Ethanol	5 min
100% Ethanol	5 min
90% Ethanol	5 min
80% Ethanol	5 min
70% Ethanol	5 min
Distilled water	5 min
Distilled water	5 min

Weigert`s Iron-hematoxylin	1 - 2 min
Water	10 min
Ponceau-acidic fuchsin -azophloxin	5 min
1% Acetic acid	rinsing
Wolfram phosphoric acid orange	5 min
1% Acetic acid	rinsing
light green	5 min
1% Acetic acid	5 min

100% Ethanol	dipping
100% Ethanol	dipping
Xylene	5 min
Xylene	5 min
Xylene	5 min

Supplement G – TRPC6 immunohistochemistry

E.1 – Peptide control

The peptide control was used to demonstrate specificity of the primary antibody. Therefore, the primary i.e. anti-TRPC6 antibody was pre-incubated with the TRPC6 blocking peptide provided by the manufacturer of the primary antibody, also. The resulting solution was utilized once instead of the pure primary antibody for establishment of the TRPC6-immunohistochemistry. The peptide control was rated as *successful* in case the slide was significantly less stained or even not stained.

E.2 – Negative control per staining run

A negative control was included in each staining run to allow for verification of the the response produced by the secondary antibody coupled with a horseradish peroxidase-coupled secondary antibody. Therefore, a diluted rabbit serum (Thermo Fisher Scientific, Carlsbad, United States of America) with a similar protein concentration as that of the primary antibody was applied instead of the antibody.

E.3 – TRPC6 immunohistochemistry

An indirect immunohistochemistry method was selected.

First the slides underwent kerosene detachment. Subsequently, for antigen recovery the sections were placed in citrate buffer at 95 degrees celsius (one hour). A normal goat serum (Ref.: G6767; Sigma Aldrich, St. Louis, Missouri, United States of America) was applied for blocking (room temperature). The primary antibodies incubated overnight. Afterwards, the secondary antibody was applied, then the chromogen diaminobenzidine. Hematoxylin according to Ehrlich (Carl Roth, Karlsruhe, Germany) was subsequently used for counterstaining.

E.4 – Selection of primary antibody concentration prior to study

Before all tissue slices were undertaken TRPC6-immunohistochemistry a serial staining with a dilution series of the primary antibody concentration was performed. Therefore, the tissue of the right atrial appendage of donor 4 was chosen as this was one of the largest samples obtained. The primary antibody was applied in the following concentrations: (1) 1:100, (2) 1:250, (3) 1:500, and (4) 1:1000. The rest of the protocol remained unchanged. The overall best result was achieved with the 1:250 dilution. So, this was applied to stain the rest of the tissue slices and was used to conduct the presented study.

Supplement H – Material, chemicals, software

Table H.1 – Summary of material, chemicals / reagents, and software used

Resource (i. e. material, chemical, etc.)	Details
Primary antibody (Anti-TRPC6 Antibody)	Ref.: ACC017; Alomone Labs, Jerusalem, Israel
Secondary antibody (Anti-rabbit coupled with horseradish peroxidase)	Ref.: A10547; Invitrogen, Carlsbad, California, United States of America.
Normal rabbit serum	Ref.: PLN5001; Life Technologies, Carlsbad, California, Unites States of America.
TRPC6 Blocking Peptide	Ref.: BLP-CC017; Alomone Labs, Jerusalem, Israel
Normal goat serum	Ref.: G6767; Sigma Aldrich, St. Louis, Missouri, United States of America
alcian blue solution	Ref.: 3082; Carl Roth, Karlsruhe, Germany
citrate buffer	Ref.: ab93678; abcam, Cambridge, United Kingdom
3,3‘-diaminobenzidine	Ref.: SK-4103; Vector Laboratories, Burlingame, California, United States of America
Eosin	Obtained as ready to use solution from the central chemical stock of the Saarland University, Campus Homburg, Homburg (Saar), Germany

Table H.1 continued

Acetic acid	VWR International GmbH, Darmstadt, Germany
Ethanol	Carl Roth, Karlsruhe, Germany
Masson Goldner Trichrom	Ref.: 12043; Süsse Labortechnik, Gudensberg, Germany
Ready to use fixation solution according to Weigner (applied for body donor)	Ref.: 27796; Otto Fischar GmbH & Co. KG, Saarbrücken, Germany
Ready to use formaldehyde fixation solution (applied for body donors)	Ref.: 27244; Otto Fischar GmbH & Co. KG, Saarbrücken, Germany
Ready to use 4% phosphate buffered formalin solution	Roti-Histofix, Carl Roth, Karlsruhe, Germany
Hematoxylin according to Ehrlich	Carl Roth, Karlsruhe, Germany
Paraffin	Carl Roth, Karlsruhe, Germany
Phosphate buffered saline	Ref.: 1105.1, Carl Roth, Karlsruhe, Germany
Hydrogen peroxide	Ref.: 8683.4; central chemical stock of the Saarland University, Campus Homburg, Homburg (Saar), Germany
Pouring station (MPS/P2)	SLEE medical, Mainz, Germany
Embedding machine (Automatic Tissue Processor MTP)	SLEE medical, Mainz, Germany
Humidity chamber	Panalo-em, central chemical stock of the Saarland University, Campus Homburg, Homburg (Saar), Germany
Olympus BX60 microscope	Olympus, Shinjuku, prefecture Tokyo, Japan

Table H.1 continued	
Olympus D37 camera	Olympus, Shinjuku, prefecture Tokyo, Japan
Olympus cellSens Dimensions Version 1.15 Build 14760	Olympus, Shinjuku, prefecture Tokyo, Japan
Cooling plate	SLEE medical, Mainz, Germany
Microtome Microm RM 2025	Heidelberg Instruments, Heidelberg, Germany
Heating plate	VWR International GmbH, Darmstadt, Germany
Warming oven	VWR International GmbH, Darmstadt, Germany
water bath	GFL („Gesellschaft für Labortechnik“ [society for laboratory technique] mbH), Burgwedel, Germany
Dako Pen	Ref.: S200230-2; Agilent, Glostrup, Denmark
Cover slips	Central stock, Saarland University Hospital, Homburg (Saar), Germany (i. e. manufacturer / product can vary dependent on what was bought by the central stock department)
Mounting medium RotiHistoKitII	Carl Roth, Karlsruhe, Germany
Microtome blades	Leica-Microsystem, Wetzlar, Germany
Microscope slides „Superfrost Plus“	R. Langenbrinck, Emmendingen, Germany
R Version 4.1.3	R Core Team (2022). R: A Language and environment for statistical computing. R Foundation for Statistical Computing, Vienna, Austria.

Table H.1 continued

RStudio 2022.07.1+554	"Spotted Wakerobin" Release (7872775ebddc40635780ca1ed238934c3345c5de, 2022-07-22) for Windows Mozilla/5.0 (Windows NT 10.0; Win64; x64) AppleWebKit/537.36 (KHTML, like Gecko) QtWebEngine/5.12.8 Chrome/69.0.3497.128 Safari/537.36
